# Phenotypic and Genotypic Analysis of Antimicrobial Resistance among *Listeria monocytogenes* Isolated from Australian Food Production Chains

**DOI:** 10.3390/genes9020080

**Published:** 2018-02-09

**Authors:** Annaleise Wilson, Jessica Gray, P. Scott Chandry, Edward M. Fox

**Affiliations:** CSIRO Agriculture and Food, Werribee, VIC 3030, Australia; alw036@student.usc.edu.au (A.W.); Jess.gray@csiro.au (J.G.); scott.chandry@csiro.au (P.S.C.)

**Keywords:** *Listeria monocytogenes*, antimicrobial resistance, single-nucleotide polymorphism subtyping, *ermB*, *fepR*, Australia

## Abstract

The current global crisis of antimicrobial resistance (AMR) among important human bacterial pathogens has been amplified by an increased resistance prevalence. In recent years, a number of studies have reported higher resistance levels among *Listeria monocytogenes* isolates, which may have implications for treatment of listeriosis infection where resistance to key treatment antimicrobials is noted. This study examined the genotypic and phenotypic AMR patterns of 100 *L. monocytogenes* isolates originating from food production supplies in Australia and examined this in the context of global population trends. Low levels of resistance were noted to ciprofloxacin (2%) and erythromycin (1%); however, no resistance was observed to penicillin G or tetracycline. Resistance to ciprofloxacin was associated with a mutation in the *fepR* gene in one isolate; however, no genetic basis for resistance in the other isolate was identified. Resistance to erythromycin was correlated with the presence of the *ermB* resistance gene. Both resistant isolates belonged to clonal complex 1 (CC1), and analysis of these in the context of global CC1 isolates suggested that they were more similar to isolates from India rather than the other CC1 isolates included in this study. This study provides baseline AMR data for *L. monocytogenes* isolated in Australia, identifies key genetic markers underlying this resistance, and highlights the need for global molecular surveillance of resistance patterns to maintain control over the potential dissemination of AMR isolates.

## 1. Introduction

*Listeria monocytogenes* is a foodborne pathogen and the causative agent of the human infection, listeriosis. *L. monocytogenes* is widely distributed throughout the environment and has been isolated throughout the various stages of the food production and supply chain [1,2]. Incidences of listeriosis are frequently associated with the consumption of raw or ready-to-eat foodstuffs—processed meats, soft cheese, milk products, salad, and seafood [3]. Although rare, invasive listeriosis has a high fatality rate of 20–30% [4]. It is characterised by invasion of the central nervous system, blood, or placenta, causing symptoms such as meningitis, encephalitis, and septicaemia [5]. Those with increased susceptibility include the immunocompromised, elderly, pregnant women, and newborn children. *L. monocytogenes* is of additional concern to pregnant women as it has the ability to translocate into the placenta causing perinatal infection, miscarriage, and stillbirth [6,7].

Listeriosis has traditionally been treatable using a combination of a β-lactam antibiotic, such as amoxicillin, penicillin, or ampicillin, and an aminoglycoside, such as gentamycin [8]. Successful treatment with trimetroprim-sulfamethoxazole has been used to treat patients with an allergy to the reference treatment of penicillin, and tetracycline and erythromycin have been used as an alternative treatment option, including treatment of patients not responding to standard therapy [9,10]. Although *L. monocytogenes* is inherently resistant to some antibiotics—primarily cephalosporins, oxacillin, and fosfomycin—it has historically been susceptible to most antibiotics used to treat Gram-positive bacteria [11,12]. Interestingly, successful use of fosfomycin for ocular infections, which typically shows little activity in vitro, is thought to be due to the low-glucose environment [13]. However, there have been a series of reports of *L. monocytogenes* isolates resistant to one or more antibiotics, particularly in Southern and Western regions of Asia, which have serious associated public health implications [14,15,16,17].

Manifestation of antibiotic resistance has been decisively linked to the increased selective pressure caused by the extensive use of antibiotics as a growth promoter in livestock animals or in medical treatment of humans or animals [18]. The primary cellular mechanisms of resistance include active transport of the antibiotic out of the bacterial cell via efflux pumps, reduced cell membrane permeability, modification of the antibiotic target site, or inactivation of the antibiotic through enzymatic degradation [19].

The genetic basis of fluoroquinolone resistance in *L. monocytogenes* has been previously linked to overexpression of the genes *lde*, encoding an efflux pump of the major facilitator superfamily, and *fepA*, of the multidrug and toxic compound extrusion (MATE) family, which are universally found in *L. monocytogenes* [20]. Mutation to the quinolone resistance-determining regions (QRDR) of the genes encoding for DNA gyrase topoisomerase II (*gyrA*, *gyrB*) and topoisomerase IV (*parC* and *parE*) has also been reported to confer resistance to fluoroquinolones in Gram-positive bacteria [21]. Studies of *L. monocytogenes* by Lampidis et al. (2002) and Moreno et al. (2014) reported mutations in the QRDR region of *gyrA* resulting in amino acid changes—Thr84Ser, Phe88Asp/Phe88Glu, and Met117Ser/Met117Gly—associated with fluoroquinolone resistance [22,23].

Erythromycin resistance determinants, *erm* genes, encode for a ribosome methylase that methylates an adenine residue in the 23S portion of the 50S ribosomal subunit [24]. *erm* genes identified in *L. monocytogenes* (*ermA*, *ermB*, and *ermC*) have also been associated with mobile genetic elements, principally plasmid pAMβ1 and pIP501 originating in *Enterococcus faecalis* and *Streptococci agalactiae*, respectively [25]. *L. monocytogenes* has been observed to encode a plasmid-mediated trimethoprim-resistant dihydrofolate reductase (DHFR) [26]. Plasmid pIP823, carrying resistant DHFR gene *dfrD*, can be transferred by conjugative mobilisation between *L. monocytogenes, Staphylococci* spp., and *Escherichia coli*.

Tetracycline resistance in *L. monocytogenes* has been linked to mobile genetic elements carrying the tetracycline resistance genes *tetA*, *tetK*, and *tetL*, which confer proton antiporter efflux pump proteins, as well as *tetM* and *tetS*, conferring ribosomal protection proteins [8,27]. The most common determinant for tetracycline resistance, *tetM*, has been found in *Tn*916/*Tn*1545-like conjugative transposons first discovered in *E. faecalis* [28,29]. Poyart-Salmeron et al. (1992) identified *tetM* and *int-Tn*, an integrase required for conjugation of the *Tn*916/*Tn*1545-like transposon, in 24 of 33 *L. monocytogenes* isolates resistant to tetracycline [30]. An additional tetracycline-resistant isolate in this study harboured plasmid pIP813, also believed to have originated in *Enterococci* and *Streptococci* spp., which carried the *tetL* gene. The presence of the mobile genetic elements originating from unrelated bacterial species suggests that both Gram-negative and Gram-positive bacteria may have a significant role in resistance gene acquisition by *L. monocytogenes* [31].

To the authors’ knowledge, this is the first study to evaluate antibiotic sensitivities of *L. monocytogenes* isolated from food sources in Australia and investigated the prevalence of genetic markers associated with antimicrobial resistance (AMR) to antibiotics including the genetic basis for resistance phenotypes observed. This study characterised 100 *L. monocytogenes* isolates from Australian food production chains to establish a baseline that can be used for further AMR surveillance relating to this important human pathogen.

## 2. Materials and Methods

### 2.1. Bacterial Strains

A total of 100 *L. monocytogenes* samples encompassing isolates from dairy products (*n* = 52), meat products (*n* = 42), vegetables (*n* = 2), food (*n* = 2), dairy farm environment (*n* = 1), and seafood (*n* = 1) were recovered from Australian food production chains between 1988 and 2016. An additional 72 clonal complex 1 isolates (CC1) were included in the phylogenetic analysis of this subgroup, as defined in the standard scheme [32]. A complete list of isolates is presented in Supplementary Table S1.

### 2.2. Serotyping of PCR Serogrouping of Isolates

Isolates were differentiated by serotype using a combination of O-antigen antisera testing (Denka Seiken Co., Ltd., Tokyo, Japan) and multiplex PCR serogrouping [33].

### 2.3. Pulsed-Field Gel Electrophoresis Subtyping

Molecular subtyping of the isolates was performed using the PulseNet standard method for *L. monocytogenes* [34]. Similarity analysis of *Apa*I and *Asc*I restriction profiles was performed using BioNumerics software, v7.6 (Applied Maths, Sint-Martens-Latem, Belgium). Restriction enzyme fingerprints were analysed using the Unweighted Pair Group Method with Arithmetic Mean, Dice correlation coefficient, and a tolerance and optimisation margin of 1%. Indistinguishable isolates were defined as those that demonstrated no band differences from each other.

### 2.4. Antibiotic Susceptibility Testing

The sensitivity of all 100 isolates in this study to a panel of four antibiotics was determined, comprising ciprofloxacin (0.002–32 µg/mL), erythromycin (0.016–256 µg/mL), penicillin G (0.002–32 µg/mL), and tetracycline (0.016–256 µg/mL). Penicillin G and other β-lactam antibiotics are the foremost treatment option for *L. monocytogenes* infection [35]. Ciprofloxacin, a fluoroquinolone antibiotic, has been used to treat listeriosis with associated meningitis, although questions have been raised regarding its suitability [36]. Erythromycin, a macrolide antibiotic, and tetracycline, a polyketide, have been traditionally used as a treatment option for *L. monocytogenes* in the case of β-lactam allergy [35]. The Minimum Inhibitory Concentration (MIC) of each antimicrobial was quantitated using Oxoid M.I.C.Evaluator™ strips (Oxoid, Basingstoke, UK) on Mueller-Hinton agar plates supplemented with 5% sheep blood (Oxoid), according to the manufacturer’s instructions. Mueller-Hinton agar plates were inoculated with a bacterial solution suspension in 3 mL of Maximum Recovery Diluent adjusted to the turbidity of a 0.5 McFarland. The inoculated plates were incubated at 37 °C for 48 h. Control strains used were *Staphylococcus aureus* ATCC 29213 and *Streptococcus pneumoniae* ATCC 49619. The MIC values were interpreted using the Clinical and Laboratory Standards Institute (CLSI) or European Committee on Antimicrobial Susceptibility Testing (EUCAST) breakpoint guidelines for *L. monocytogenes* where available, and *Staphylococci* spp. [37,38].

### 2.5. Whole Genome Sequencing and Multilocus Sequence Typing 

Draft genomes for each isolate were subjected to bioinformatic analyses. Draft genomes were prepared as previously described [39]. Gene BLAST searches and alignments of *L. monocytogenes* isolates tested in this study were performed using the Geneious software platform v10.0.7 [40]. Multilocus Sequence Typing (MLST) STs were determined by examining the gene sequence of seven housekeeping genes, using primers and gene targets previously described [32]. Isolates included representatives of major *L. monocytogenes* sequence types (STs) previously reported circulating in Australia, such as ST1, ST2, ST3, ST155, and ST204 [41]. A list of AMR genes used in BLAST searches is presented in Table S2.

### 2.6. Phylogenetic Analysis of Clonal Complex 1 Isolates

The single-nucleotide polymorphism (SNP) phylogeny was determined with kSNP v3.1 [42] for 79 isolates using a mixture of finished genomes, draft genomes, and read data. Paired-end read files were quality filtered, converted to fasta and merged using seqtk v1.2–r102 (https://github.com/lh3/seqtk). The core phylogeny was determined using a Kmer setting of 21 as determined with the Kchooser program in kSNP v3.1.

## 3. Results

### 3.1. Serotyping

*L. monocytogenes* may be classified into genetic lineages [43]. O-antigen antiserum combined with multiplex PCR serotyping identified that 56 isolates were of genetic lineage II (1/2a, 3a, 1/2c, and 3c); the majority were associated with serotype 1/2a (*n* = 48), followed by serotype 1/2c (*n* = 4), and serotype 3a (*n* = 4). Forty-three isolates were determined to be of Lineage I (1/2b, 3b, 7, 4b, 4d, and 4e); the majority were identified as serotype 1/2b (*n* = 30), followed by 4b (*n* = 8), 3b (*n* = 3), and 4d (*n* = 2). Only one Lineage III isolate was identified, which was serotype 4a.

### 3.2. Pulsed-Field Gel Electrophoresis Subtyping

**Figure 1:**
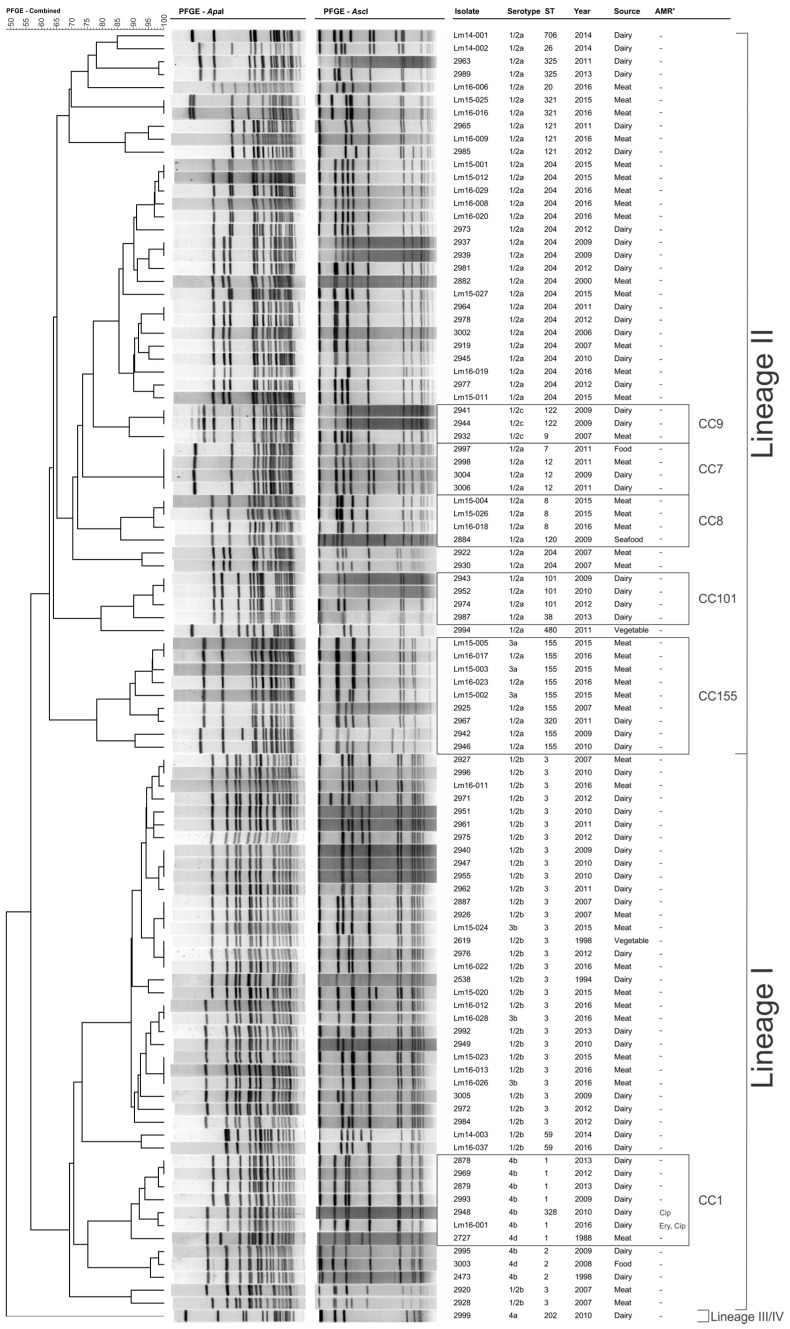
Pulsed-Field Gel Electrophoresis dendrogram of isolates in this study. ^a^AMR, antimicrobial resistance; CC, clonal complex; Cip, ciprofloxacin resistance; Ery, erythromycin resistance; ST: sequence type. Restriction enzyme fingerprints were analysed using the Unweighted Pair Group Method with Arithmetic Mean, Dice correlation coefficient, and a tolerance and optimisation margin of 1%. Percentage similarity is shown on the upper-left scale.

Analysis of the combined *Apa*I and *Asc*I identified 78 unique pulsotypes (Figure 1). Isolates were clustered into the three groups which related to Lineage I, Lineage II, and Lineages III/IV.

### 3.3. Antimicrobial Resistance Characteristics

**Figure 2:**
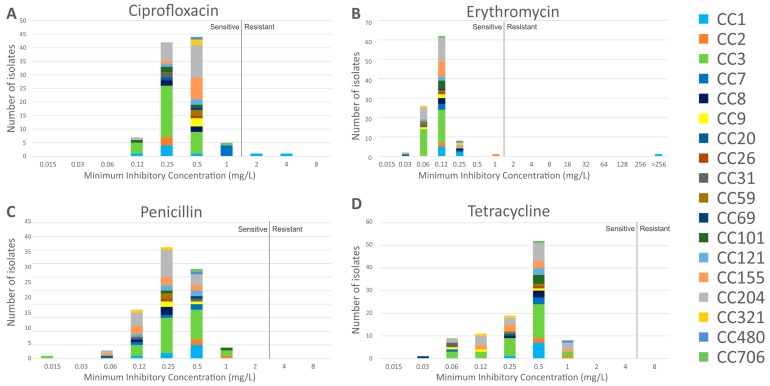
Minimum Inhibitory Concentration (MIC) values of isolates in this study to the panel of four antibiotics. Breakpoints are indicated by a vertical black line in each graph; MICs to the left of this are considered sensitive, while those to the right are considered resistant. Breakpoints (mg/L): Ciprofloxacin S ≤ 1, R > 1; Erythromycin S ≤ 1, R > 1; Penicillin S ≤ 2, R > 2; Tetracycline S ≤ 4, R > 4. CC, clonal complex.

The MIC distribution of the isolates to the panel of four antibiotics is shown in Figure 2. Susceptibility was defined as a MIC value of ≤1 mg/L for ciprofloxacin (EUCAST *S. aureus* breakpoint, as no *L. monocytogenes* breakpoint is defined), ≤1 mg/L for erythromycin (EUCAST breakpoint), ≤2 mg/L for penicillin G (CLSI breakpoint), and ≤4 mg/L for tetracycline (CLSI *S. aureus* breakpoint, as no *L. monocytogenes* breakpoint is defined). All isolates were found to be susceptible to penicillin G and tetracycline. Two serotype 4b isolates were resistant to ciprofloxacin only (2948 and Lm16-001, MIC of 2 mg/L and 4 mg/L, respectively); isolate Lm16-001 was also resistant to erythromycin (MIC > 256 mg/L).

### 3.4. Detection of Genetic Markers Related to Antimicrobial Resistance

All isolates were found to harbour the fosfomycin resistance gene, *fosX*, and the lincomycin resistance gene, *lmrB*. An analysis of the QRDR regions of *gyrA*, *gyrB*, *parC*, and *parE* genes revealed no mutations responsible for fluoroquinolone resistance. There were no tetracycline (*tetA*, *tetK*, *tetL*, *tetM* and *tetS*), trimethoprim (*dfrD* and *dfrG*), or vancomycin (*vanA* and *vanB*) resistance-associated genes identified. No mobile genetic elements containing antibiotic resistance determinants were detected. Interrogation of erythromycin resistance determinants (*ermA*, *ermB*, and *ermC*) identified a single resistance gene, *ermB*, harboured by isolate Lm16-001. This correlated with the phenotypic observation of erythromycin resistance of this isolate (>256 mg/L).

**Figure 3:**
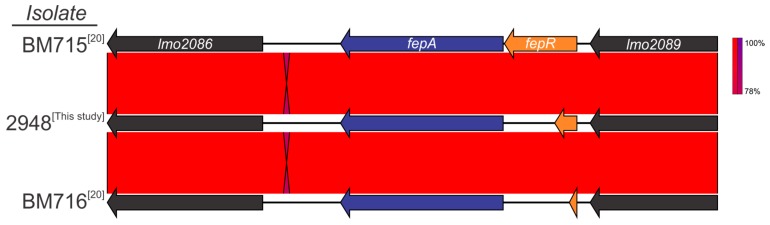
Comparison of a full-length wild-type *fepR* protein with the truncated *fepR* protein identified in this study and that of Guerin et al. 2014 [20] conferring resistance to ciprofloxacin.

When comparing the genome of ciprofloxacin-resistant (Cip^R^) isolate 2948 to wild-type (WT) isolates, a single-point mutation was identified in the fluoroquinolone efflux protein (*fepA*) regulator, *fep*R (Figure 3). The mutation, located at nucleotide position 181, resulted in a premature stop codon (PMSC) in place of amino acid aspartic acid (E61*). Analysis of the *fepR* gene in the other ciprofloxacin resistant isolate (Lm16-001) did not identify a mutation at this position, with this isolate encoding a full length *fepR* protein. However, a unique single-point mutation at nucleotide position 170 caused an amino acid change from alanine to glycine. Further investigation is required to determine if this has a role in the Cip^R^ phenotype of Lm16-001.

### 3.5. Clonal Complex 1 Population Structure Analysis

**Figure 4:**
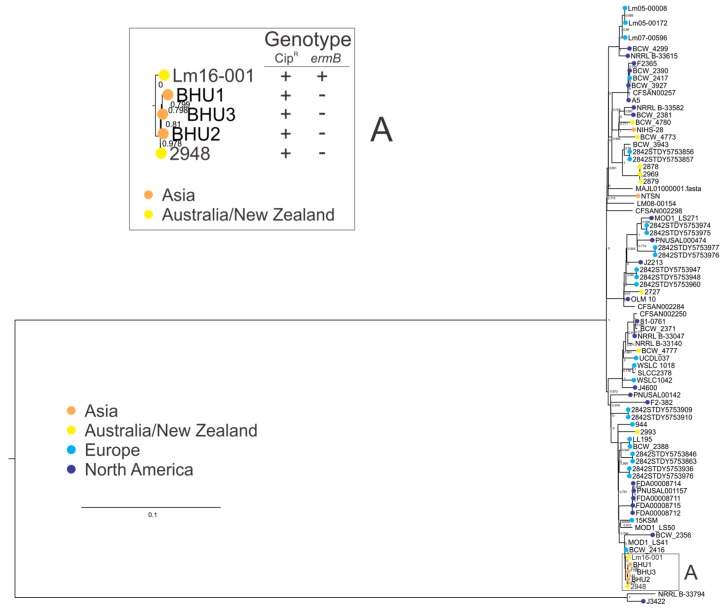
Phylogenetic analysis of clonal complex 1 (CC1) genomes, showing a parsimony tree generated by kSNP using a core single-nucleotide polymorphism (SNP) alignment. Box A: clade containing both AMR isolates identified in this study, and three other ST328 isolates from India. With the exception of Lm16-001, isolates in this clade had a mutation leading to a premature stop codon (PMSC) in their respective *fepR* genes. Genotype: Cip^R^, PMSC in the *fepR* gene and/or phenotypically demonstrated resistance (+); *ermB*, presence (+) or absence (−).

Clonal complexes are groups of closely related STs which share sequence homology among at least six alleles. Further analysis was performed on a larger set of CC1 isolates to understand the phylogeny of the CC1 isolates in this study with a set of isolates from other global regions. Analysis of the CC1 genomes (Figure 4) showed antimicrobial resistant isolates in this study clustered together, and were separate from this study’s other CC1 isolates. Expanding the analysis to include additional CC1 isolates showed both AMR isolates (2948 and Lm16-001) to be more closely related to three isolates from India (BHU1, BHU2, and BHU3; clade A in Figure 4) than to other CC1 isolates (including both other CC1 isolates from this study and other CC1 isolates from other global regions). Analysis of isolates in clade A identified a ciprofloxacin genotype and/or phenotype, suggesting that a ciprofloxacin resistance phenotype is associated with this small subgroup of *L. monocytogenes* within CC1, although it should be noted the majority of CC1 isolates in this analysis did not contain the ciprofloxacin resistance marker.

## 4. Discussion

Modern years have seen the dynamics of food production and supply shift from locally grown and consumed foods to a globally connected food supply paradigm [44]. This presents new challenges to the control of foodborne pathogens and has implications for their ecology. The capacity for strains of *L. monocytogenes* to colonise and persist in localised environments has been extensively reported [45,46,47], and, as such, globalised food chains present new vectors for *L. monocytogenes* distribution to other geographical niches. These new vectors present new opportunities for opportunistic colonisation of new niches across the globe, and, as such, present a new challenge to maintaining food safety.

Until recent years, studies on antibiotic resistance of *L. monocytogenes* cohorts have typically reported low levels of resistance; however, recent studies, including studies of isolates from Asia and the Middle East, have reported an emerging prevalence of AMR among *L. monocytogenes* isolated from those regions (Table 1). Other studies including temporal analysis of isolates from the same geographical region suggest an increasing trend in *L. monocytogenes* AMR, although, importantly, this resistance has not typically been to the key treatment choices for listeriosis infection [48,49]. This may have implications for public health in these regions regarding treatment of listeriosis, should resistance to key treatments evolve and expand, but more broadly has implications for dissemination of AMR isolates across the globe through contaminated food carriage since this represents the primary vector for human transmission [50].

The findings of this study suggest that the overall levels of antibiotic resistance among *L. monocytogenes* isolates from food production systems in Australia is relatively low. Despite this, emerging reports of resistant strains of *L. monocytogenes* to reference or alternative antibiotic treatments could present a public health risk due to the high mortality rate of this human pathogen. To this end, further investigation of the molecular ecology of resistant isolates can not only yield insights into the geographical distribution of these resistant isolates, but also provide valuable baseline data to build an ongoing surveillance platform to monitor AMR among target species such as *L. monocytogenes*. This analysis can also identify key resistance markers which contribute to this resistance, and examine their implications for dissemination through the population. While genetic mutations such as SNPs giving rise to a resistance phenotype do not necessarily have serious implications for transfer through the bacterial population, the occurrence of resistance markers on transferrable mobile genetic elements such as transposons or plasmids may have significant implications for dissemination of resistance.

This study found no isolates that were resistant to penicillin G or tetracycline. A single isolate was resistant to erythromycin. Increased rates of resistance to erythromycin have been reported in Indonesia (6.9%), Iran (14%), Yemen (58.8%), Egypt (62.5%), and India (85.7%) [17,33,57]. The erythromycin resistant isolate in this study harboured the *ermB* gene, and displayed high resistance to the antibiotic (>256 mg/L). MLST subtyping identified this isolate as an ST1 isolate; however, this isolate showed closer genotypic homology to ST328 isolates than to other ST1 isolates in this analysis (Figure 1). It should be noted that both ST1 and ST328 are closely related members of the *L. monocytogenes* CC1. Similar clustering results were observed when additional CC1 isolates were included in a phylogenetic analysis (Figure 4); additional CC1 isolates from other global regions will provide greater insight into the evolution of CC1. Isolates from the ST328 subgroup are among the most prevalent of both clinical and food isolates in India. While this dataset does not include epidemiological information relating to the foods from which these strains were isolated, nonetheless, ongoing surveillance of import and export foods clearly has a place in food safety programs. Indeed, the debate regarding use of antibiotics and associated regulations in food animals is ongoing [63], and, as such, understanding food contamination and transmission of key pathogens is becoming an increasingly important facet of food safety protocol. It is worth noting that the high prevalence of AMR noted by Obaidat et al. (2015) was among *L. monocytogenes* from export foods, again highlighting this emerging food safety issue [34].

Resistance to ciprofloxacin was identified in only 2% (*n* = 2) of isolates in this study. Reports of ciprofloxacin resistance in *L. monocytogenes* have been consistently low with the exception of a study in China that observed a frequency of 17.8% [15]. Ciprofloxacin is not traditionally used as a treatment option in cases of listeriosis. As such, the prevalence of resistant strains in this study is not of critical significance in the context of current treatment courses from a public health perspective. A SNP mutation (G61T) in the fluoroquinolone efflux pump repressor gene, *fepR*, was previously identified as the mechanism of ciprofloxacin resistance in a clinical *L. monocytogenes* isolate [20].

Through the construction of a deletion mutant in a WT *L. monocytogenes* strain, the researchers were able to confirm the role of *fepR* as a transcriptional repressor of *fepA* and observed a 32-fold increase in MIC value to ciprofloxacin in the deletion mutant. A similar mutation was identified in the ciprofloxacin-resistant isolate 2948 in this study. However, the mutation found in strain 2948 in this study (G181T) was located 120 bp downstream of that described in the previous study (Figure 3). Restoration of the full-length *fepR* gene is nonetheless required to confirm its role in the cirpfloxacin phenotype of isolate 2948. The *fepR* gene of Lm16-001, in contrast, was identified as a full-length genotype. In addition, no mutations known to impart AMR were identified in the *gyrA* or *parC* genes of Lm16-001. As such, further study is needed to identify the underlying mechanism for ciprofloxacin resistance in this isolate.

The high similarity evidenced by the PFGE profiles of isolates 2948 and Lm16-001 suggested that they may be genetically similar. Combined PFGE and MLST analysis indicated that the resistant isolate Lm16-001 was ST1 and more closely related to the ST328 resistant isolate, rather than the other ST1 isolates in this study; this observation underlies the need for increased datasets including additional CC1 isolates to provide a stronger understanding of the true distribution of CC1 clones across Australasia and, indeed, globally. Both isolates belong to serotype 4b, which causes the majority of human listeriosis cases [50]. Interestingly, *L. monocytogenes* MLST type ST328 is rarely isolated in Australia, where ST1, ST3, and ST204 dominate [40,42]. MLST type ST328 is highly abundant among strains isolated in India [64]. Further analysis of CC1 isolates from different global regions showed both resistant isolates in this study clustered closely with the isolates from India (clade A, Figure 4). Although data is limited, some studies of *L. monocytogenes* isolated from India have reported high frequencies of resistance to penicillin (100%), tetracycline (90.5%), and erythromycin (85.7%) [62,33]. Also concerning is the finding of vancomycin resistance (57.7%) among isolates, as this antibiotic is considered a last line of defense against multidrug-resistant Gram-positive bacteria [65]. Further work is required to understand if these emerging reports of AMR are more widely distributed across the *L. monocytogenes* population or limited to the subsets of isolates in the associated studies. All isolates in clade A demonstrated genotypic (contained a PMSC in their *fepR* genes) or phenotypic ciprofloxacin resistance, suggesting that the isolates from India in this clade may also be phenotypically ciprofloxacin resistant; however, it should be noted that data in this study does not suggest that ciprofloxacin resistance is widely distributed among CC1 isolates.

## 5. Conclusions

There currently exists insufficient breakpoint documentation required for MIC interpretation, impeding standardised 4ave established breakpoints for ampicillin, benzylpenicillin, erythromycin, meropenem, penicillin, and Trimethoprim-sulfamethoxazole only [54,55]. Furthermore, there is a lack of empirical data in Australia on AMR in foodborne *L. monocytogenes* in order to establish the significance of the resistance levels reported in this study. Further surveillance in Australia of the prevalence of AMR in this important human pathogen is recommended; however, this study provides a basis for further investigation. National antimicrobial stewardship of food animals is an important aspect of the One Health approach to control AMR [66]. Findings of this study support the need for a global context approach to AMR surveillance of food chains. National AMR surveillance programs must not rely solely on breakpoint data, but should include population analysis through molecular subtyping to monitor resistant subpopulations and potential emerging resistant subgroups. In addition, the role of food imports and exports on the dissemination of AMR requires consideration.

## Figures and Tables

**Table 1:** Previous studies documenting antimicrobial resistance (AMR) among *Listeria monocytogenes* isolate cohorts.

Location	Collection Start Date	Sample Size	Ciprofloxacin (% Resistant)	Erythromycin (% Resistant)	Penicillin (% Resistant)	Tetracycline (% Resistant)	Study
Brazil	1978	26	7.1	0	0	0	[22]
France	1989	4668	0.43	0.02	NT ^a^	0.7	[48]
Brazil	2003	50	0	6	0	2	[51]
China	2005	90	17.8	2.22	1.11	15.6	[15]
USA	2005	157	3	0	0	3	[52]
Switzerland	2006	383	7	0	0	1.3	[29]
Ireland	2007	191	0	0	4	1	[53]
Italy	2008	120	1.7	0	0	0.8	[54]
USA	2009	90	2	0	0	1	[55]
Ireland	2011	51	0	0	0	0	[1]
Iran	2012	43	NT	14	16.3	27.9	[56]
Ethiopia	2012	24	NT	NT	66.7	37.5	[57]
Indonesia	2012	29	0	6.9	17.2	0	[17]
China	2013	78	0	1.3	1.3	20.5	[58]
Colombia	2013	259	7.4	15.8	0	6.6	[59]
Turkey	2014	12	0	NT	66.7	0	[60]
India	2014	5	0	NT	100	20	[61]
Yemen	2015	51	NT	58.8	100	56.8	[62]
India	2015	21	NT	85.7	100	90.5	[62]
Egypt	2015	32	NT	62.5	93.8	59.4	[62]

^a^ NT, Not tested.

## Supplementary Materials

The following are available online at http://www.mdpi.com/2073-4425/9/2/80/s1.
