# Supplementary material for: Phenotypic and Genotypic Analysis of Antimicrobial Resistance among Listeria monocytogenes Isolated from Australian Food Production Chains
Source: Genes (Basel). 2018 Feb 9;9(2):80. doi: 10.3390/genes9020080 (PMC5852576; doi:10.3390/genes9020080)
Supplement: Supplementary file 1 [file genes-09-00080-s001.zip › Figure S1.docx]

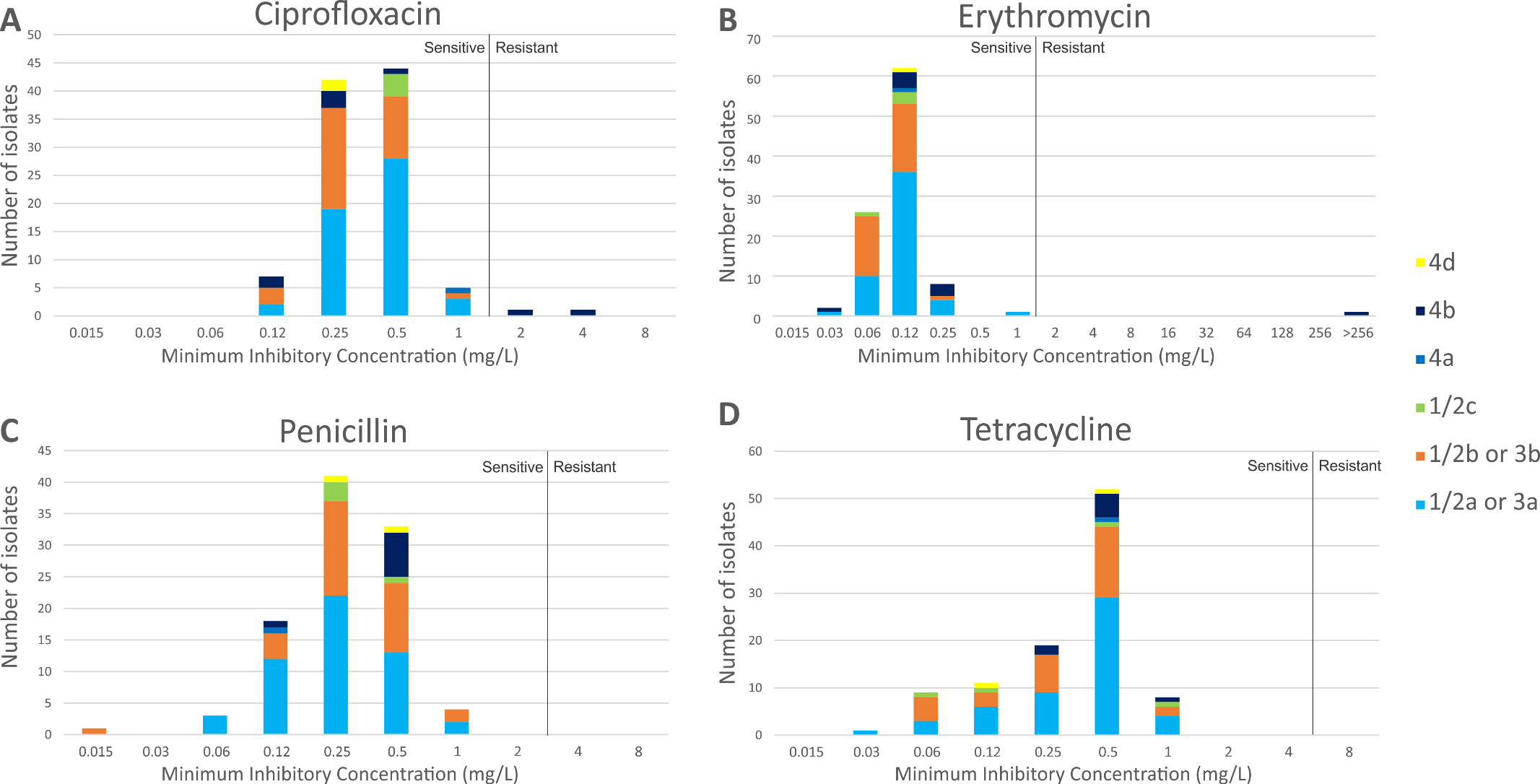


**Figure S1.** MIC values of isolates in this study to the panel of 4 antibiotics. Breakpoints are indicated by a vertical black line in each graph; MICs to the left of this considered sensitive, while those to the right considered resistant. Breakpoints (mg/L): Ciprofloxacin S ≤1, R>1; Erythromycin S ≤1, R>1; Penicillin S ≤2, R>2; Tetracycline S ≤4, R>4.
