# Supplementary material for: Phenotypic and Genotypic Analysis of Antimicrobial Resistance among Listeria monocytogenes Isolated from Australian Food Production Chains
Source: Genes (Basel). 2018 Feb 9;9(2):80. doi: 10.3390/genes9020080 (PMC5852576; doi:10.3390/genes9020080)
Supplement: Supplementary file 1 [file genes-09-00080-s001.zip › Table S1 - Revised.docx]

**Supplementary Table 1** *L. monocytogenes* isolates included in this study

| Isolate | | Associated Food Supply | Country | Date | Serotype | Clonal Complex | GenBank Accession Number | | |
| --- | --- | --- | --- | --- | --- | --- | --- | --- | --- |
| 2878 | | Dairy | Australia | 2013 | 4b | 1 |  | |  |
| 2879 | | Dairy | Australia | 2013 | 4b | 1 |  | |  |
| 2948 | | Dairy | Australia | 2010 | 4b | 1 |  | |  |
| 2969 | | Dairy | Australia | 2012 | 4b | 1 |  | |  |
| 2993 | | Dairy | Australia | 2009 | 4b | 1 |  | |  |
| Lm16-001 | | Dairy | Australia | 2016 | 4b | 1 |  | |  |
| 2727 | | Meat | Australia | 1988 | 4d | 1 | NAVA00000000 | |  |
| 2473 | | Dairy | Australia | 1998 | 4b | 2 |  | |  |
| 2995 | | Dairy | Australia | 2009 | 4b | 2 |  | |  |
| 3003 | | Mixed Food | Australia | 2008 | 4d | 2 |  | |  |
| 2538 | | Dairy | Australia | 1994 | 1/2b | 3 |  | |  |
| 2619 | | Vegetable | Australia | 1998 | 1/2b | 3 |  | |  |
| 2887 | | Dairy | Australia | 2007 | 1/2b | 3 | NAUZ00000000 | |  |
| 2920 | | Meat | Australia | 2007 | 1/2b | 3 |  | |  |
| 2926 | | Meat | Australia | 2007 | 1/2b | 3 |  | |  |
| 2927 | | Meat | Australia | 2007 | 1/2b | 3 |  | |  |
| 2928 | | Meat | Australia | 2007 | 1/2b | 3 |  | |  |
| 2940 | | Dairy | Australia | 2009 | 1/2b | 3 |  | |  |
| 2947 | | Dairy | Australia | 2010 | 1/2b | 3 | NAUV00000000 | |  |
| 2949 | | Dairy | Australia | 2010 | 1/2b | 3 |  | |  |
| 2951 | | Dairy | Australia | 2010 | 1/2b | 3 |  | |  |
| 2955 | | Dairy | Australia | 2010 | 1/2b | 3 | NAUY00000000 | |  |
| 2961 | | Dairy | Australia | 2011 | 1/2b | 3 | NAUX00000000 | |  |
| 2962 | | Dairy | Australia | 2011 | 1/2b | 3 | NAUW00000000 | |  |
| 2971 | | Dairy | Australia | 2012 | 1/2b | 3 | NAUU00000000 | |  |
| 2972 | | Dairy | Australia | 2012 | 1/2b | 3 | NAUT00000000 | |  |
| 2975 | | Dairy | Australia | 2012 | 1/2b | 3 | NAUS00000000 | |  |
| 2976 | | Dairy | Australia | 2012 | 1/2b | 3 | NAUR00000000 | |  |
| 2984 | | Dairy | Australia | 2012 | 1/2b | 3 |  | |  |
| 2992 | | Dairy | Australia | 2013 | 1/2b | 3 |  | |  |
| 2996 | | Dairy | Australia | 2010 | 1/2b | 3 |  | |  |
| 3005 | | Dairy | Australia | 2009 | 1/2b | 3 |  | |  |
| Lm15-020 | | Meat | Australia | 2015 | 1/2b | 3 |  | |  |
| Lm15-023 | | Meat | Australia | 2015 | 1/2b | 3 |  | |  |
| Lm16-011 | | Meat | Australia | 2016 | 1/2b | 3 |  | |  |
| Lm16-012 | | Meat | Australia | 2016 | 1/2b | 3 |  | |  |
| Lm16-013 | | Meat | Australia | 2016 | 1/2b | 3 |  | |  |
| Lm16-022 | | Meat | Australia | 2016 | 1/2b | 3 |  | |  |
| Lm15-024 | | Meat | Australia | 2015 | 3b | 3 |  | |  |
| Lm16-026 | | Meat | Australia | 2016 | 3b | 3 |  | |  |
| Lm16-028 | | Meat | Australia | 2016 | 3b | 3 |  | |  |
| 2997 | | Mixed Food | Australia | 2011 | 1/2a | 7 |  | |  |
| 2884 | | Seafood | Australia | 2009 | 1/2a | 8 | NAUN00000000 | |  |
| Lm15-004 | | Meat | Australia | 2015 | 1/2a | 8 |  | |  |
| Lm15-026 | | Meat | Australia | 2015 | 1/2a | 8 |  | |  |
| Lm16-018 | | Meat | Australia | 2016 | 1/2a | 8 |  | |  |
| 2932 | | Meat | Australia | 2007 | 1/2c | 9 |  | |  |
| 2941 | | Dairy | Australia | 2009 | 1/2c | 9 | NAUP00000000 | |  |
| 2944 | | Dairy | Australia | 2009 | 1/2c | 9 | NAUO00000000 | |  |
| 2998 | | Meat | Australia | 2011 | 1/2a | 12 |  | |  |
| 3004 | | Dairy | Australia | 2009 | 1/2a | 12 |  | |  |
| 3006 | | Dairy | Australia | 2011 | 1/2a | 12 |  | |  |
| Lm16-006 | | Meat | Australia | 2016 | 1/2a | 20 |  | |  |
| Lm14-002 | | Dairy | Australia | 2014 | 1/2a | 26 |  | |  |
| Lm14-003 | | Dairy | Australia | 2014 | 1/2b | 59 |  | |  |
| Lm16-037 | | Dairy | Australia | 2016 | 1/2b | 59 |  | |  |
| 2943 | | Dairy | Australia | 2009 | 1/2a | 101 | NAUH00000000 | |  |
| 2952 | | Dairy | Australia | 2010 | 1/2a | 101 | NAUI00000000 | |  |
| 2974 | | Dairy | Australia | 2012 | 1/2a | 101 |  | |  |
| 2987 | | Dairy | Australia | 2013 | 1/2a | 101 | NAUJ00000000 | |  |
| 2965 | | Dairy | Australia | 2011 | 1/2a | 121 |  | |  |
| 2985 | | Dairy | Australia | 2012 | 1/2a | 121 | NAUE00000000 | |  |
| Lm16-009 | | Meat | Australia | 2016 | 1/2a | 121 |  | |  |
| 2925 | | Meat | Australia | 2007 | 1/2a | 155 |  | |  |
| 2942 | | Dairy | Australia | 2009 | 1/2a | 155 | NAUM00000000 | |  |
| 2946 | | Dairy | Australia | 2010 | 1/2a | 155 | NAUL00000000 | |  |
| 2967 | | Dairy | Australia | 2011 | 1/2a | 155 | NAUK00000000 | |  |
| Lm16-017 | | Meat | Australia | 2016 | 1/2a | 155 |  | |  |
| Lm16-023 | | Meat | Australia | 2016 | 1/2a | 155 |  | |  |
| Lm15-002 | | Meat | Australia | 2015 | 3a | 155 |  | |  |
| Lm15-003 | | Meat | Australia | 2015 | 3a | 155 |  | |  |
| Lm15-005 | | Meat | Australia | 2015 | 3a | 155 |  | |  |
| 2999 | | Dairy | Australia | 2010 | 4a | 202 |  | |  |
| 2882 | | Meat | Australia | 2000 | 1/2a | 204 | LXQP00000000 | |  |
| 2919 | | Meat | Australia | 2007 | 1/2a | 204 | LXQQ00000000 | |  |
| 2922 | | Meat | Australia | 2007 | 1/2a | 204 |  | |  |
| 2930 | | Meat | Australia | 2007 | 1/2a | 204 |  | |  |
| 2937 | | Dairy | Australia | 2009 | 1/2a | 204 | LXQR00000000 | |  |
| 2939 | | Dairy | Australia | 2009 | 1/2a | 204 | LXQS00000000 | |  |
| 2945 | | Dairy | Australia | 2010 | 1/2a | 204 | LXQT00000000 | |  |
| 2964 | | Dairy | Australia | 2011 | 1/2a | 204 | LXQU00000000 | |  |
| 2973 | | Dairy | Australia | 2012 | 1/2a | 204 | LXQV00000000 | |  |
| 2977 | | Dairy | Australia | 2012 | 1/2a | 204 | LXQW00000000 | |  |
| 2978 | | Dairy | Australia | 2012 | 1/2a | 204 | LXQX00000000 | |  |
| 2981 | | Dairy | Australia | 2012 | 1/2a | 204 | LXQY00000000 | |  |
| 3002 | | Dairy | Australia | 2006 | 1/2a | 204 | LXQZ00000000 | |  |
| Lm15-001 | | Meat | Australia | 2015 | 1/2a | 204 | LXRA00000000 | |  |
| Lm15-011 | | Meat | Australia | 2015 | 1/2a | 204 | LXRB00000000 | |  |
| Lm15-012 | | Meat | Australia | 2015 | 1/2c | 204 |  | |  |
| Lm15-027 | | Meat | Australia | 2015 | 1/2a | 204 | LXRC00000000 | |  |
| Lm16-019 | | Meat | Australia | 2016 | 1/2a | 204 |  | |  |
| Lm16-020 | | Meat | Australia | 2016 | 1/2a | 204 |  | |  |
| Lm16-029 | | Meat | Australia | 2016 | 1/2a | 204 |  | |  |
| Lm16-008 | | Meat | Australia | 2016 | 3a | 204 |  | |  |
| Lm15-025 | | Meat | Australia | 2015 | 1/2a | 321 |  | |  |
| Lm16-016 | | Meat | Australia | 2016 | 1/2a | 321 |  | |  |
| 2963 | | Dairy | Australia | 2011 | 1/2a | 325 |  | |  |
| 2989 | | Dairy | Australia | 2013 | 1/2a | 325 |  | |  |
| 2994 | | Vegetable | Australia | 2011 | 1/2a | 480 |  | |  |
| Lm14-001 | | Dairy | Australia | 2014 | 1/2a | 706 |  | |  |
| 15KSM | |  | Austria | 2013 |  | 1 | JYOT01000024 | |  |
| 2842STDY5753856 | |  | Netherlands | 2006/2012 |  | 1 | FFEO01000001 | |  |
| 2842STDY5753857 | |  | Netherlands | 2006/2012 |  | 1 | FFGJ01000001 | |  |
| 2842STDY5753863 | |  | Netherlands | 2006/2012 |  | 1 | FFER01000002 | |  |
| 2842STDY5753864 | |  | Netherlands | 2006/2012 |  | 1 | FFGL01000002 | |  |
| 2842STDY5753909 | |  | Netherlands | 2006/2012 |  | 1 | FFFD01000003 | |  |
| 2842STDY5753910 | |  | Netherlands | 2006/2012 |  | 1 | FFHA01000002 | |  |
| 2842STDY5753936 | |  | Netherlands | 2006/2012 |  | 1 | FFFJ01000004 | |  |
| 2842STDY5753937 | |  | Netherlands | 2006/2012 |  | 1 | FFHH01000004 | |  |
| 2842STDY5753947 | |  | Netherlands | 2006/2012 |  | 1 | FFFT01000004 | |  |
| 2842STDY5753948 | |  | Netherlands | 2006/2012 |  | 1 | FFHL01000003 | |  |
| 2842STDY5753960 | |  | Netherlands | 2006/2012 |  | 1 | FFFU01000004 | |  |
| 2842STDY5753974 | |  | Netherlands | 2006/2012 |  | 1 | FFGA01000003 | |  |
| 2842STDY5753975 | |  | Netherlands | 2006/2012 |  | 1 | FFHW01000003 | |  |
| 2842STDY5753976 | |  | Netherlands | 2006/2012 |  | 1 | FFGB01000003 | |  |
| 2842STDY5753977 | |  | Netherlands | 2006/2012 |  | 1 | FFHX01000001 | |  |
| 81-0861 | |  | Canada | 1981 |  | 1 | CP006874 | |  |
| 944 | |  | Ireland | 2013 |  | 1 | LJPD01000005 | |  |
| A5 | |  | Canada | 1990 |  | 1 | MDRB01000008 | |  |
| BCW_2356 | |  | United States | 2005 |  | 1 | MJSM01000007 | |  |
| BCW_2371 | |  |  | 1981 |  | 1 | MJSY01000003 | |  |
| BCW_2381 | |  | United States | 2011 |  | 1 | MJTH01000008 | |  |
| BCW_2388 | |  | Switzerland | 1983 |  | 1 | MJTO01000003 | |  |
| BCW_2390 | |  | United States | 1985 |  | 1 | MJTQ01000014 | |  |
| BCW_2416 | |  | Switzerland | 1983 |  | 1 | MJCO01000003 | |  |
| BCW_2417 | |  | Switzerland | 1983 |  | 1 | MJCP01000003 | |  |
| BCW_3927 | |  | United States | 1985 |  | 1 | MJNZ01000004 | |  |
| BCW_3943 | |  |  |  |  | 1 | MJOQ01000003 | |  |
| BCW_4299 | |  | United States |  |  | 1 | MJQN01000003 | |  |
| BCW_4773 | |  | New Zealand | 2009 |  | 1 | MJRQ01000006 | |  |
| BCW_4777 | |  | New Zealand | 2012 |  | 1 | MJRU01000004 | |  |
| BCW_4780 | |  | New Zealand | 2012 |  | 1 | MJRW01000002 | |  |
| BHU1 | |  | India | 2014 |  | 1 | JUKE01000002 | |  |
| BHU2 | |  | India | 2014 |  | 1 | JUKF01000009 | |  |
| BHU3 | |  | India | 2014 |  | 1 | JUKG01000002 | |  |
| CFSAN002250 | |  |  |  |  | 1 | MJTM01000014 | |  |
| CFSAN002257 | |  |  |  |  | 1 | MJSJ01000015 | |  |
| CFSAN002284 | |  |  |  |  | 1 | MJTY01000002 | |  |
| CFSAN002298 | |  |  |  |  | 1 | MJCM01000007 | |  |
| F2365 | |  | United States | 1985 |  | 1 | NC_002973 | |  |
| F2-382 | |  | United States | 2005 |  | 1 | BAZC01000039 | |  |
| FDA00008711 | |  | United States | 2015 |  | 1 | LNPM01000001 | |  |
| FDA00008712 | |  | United States | 2015 |  | 1 | LNPN01000009 | |  |
| FDA00008714 | |  | United States | 2015 |  | 1 | LNPP01000005 | |  |
| FDA00008715 | |  | United States | 2015 |  | 1 | LNPQ01000002 | |  |
| FSIS1606618 | |  | United States | 2016 |  | 1 | MAJL01000001 | |  |
| J2213 | |  | United States | 2003 |  | 1 | MLFL01000016 | |  |
| J3422 | |  | United States | 2005 |  | 1 | MNCC01000019 | |  |
| J4600 | |  | United States | 2007 |  | 1 | MNCD01000011 | |  |
| LL195 | |  |  |  |  | 1 | HF558398 | |  |
| LM05-00008 | |  | France | 2005 |  | 1 | CYUP01000013 | |  |
| LM05-00172 | |  | France | 2005 |  | 1 | CYUS01000009 | |  |
| LM07-00596 | |  | France | 2007 |  | 1 | CYVI01000010 | |  |
| LM08-00154 | |  | France | 2008 |  | 1 | CYWD01000008 | |  |
| MOD1_LS271 | |  | United States | 1994 |  | 1 | LNNQ01000008 | |  |
| MOD1_LS41 | |  |  |  |  | 1 | LNOC01000006 | |  |
| MOD1_LS50 | |  |  |  |  | 1 | LNOE01000001 | |  |
| NIHS-28 | |  | Japan |  |  | 1 | BAZD01000018 | |  |
| NRRL B-33047 | |  | Canada | 1981 |  | 1 | MKMD01000003 | |  |
| NRRL B-33140 | |  |  |  |  | 1 | MKMN01000007 | |  |
| NRRL B-33582 | |  | United States | 2001 |  | 1 | MKOT01000003 | |  |
| NRRL B-33615 | |  | United States | 2003 |  | 1 | MKPB01000009 | |  |
| NRRL B-33794 | |  |  | 2005 |  | 1 | MKPI01000008 | |  |
| NTSN | |  | China | 2011 |  | 1 | CP009897 | |  |
| OLM 10 | |  | United States | 1933 |  | 1 | MIMA01000005 | |  |
| PNUSAL001142 | |  | United States | 2014 |  | 1 | LYVE01000001 | |  |
| PNUSAL001157 | |  | United States | 2014 |  | 1 | LNSL01000023 | |  |
| SLCC2378 | |  |  |  |  | 1 | FR733644 | |  |
| WSLC 1018 | |  | United Kingdom | 1961 |  | 1 | CP013285 | |  |
| WSLC 1042 | |  | Germany |  |  | 1 | CP007210 | |  |
|  |  | | | | | | |  |  |
