# Supplementary material for: Phenotypic and Genotypic Analysis of Antimicrobial Resistance among Listeria monocytogenes Isolated from Australian Food Production Chains
Source: Genes (Basel). 2018 Feb 9;9(2):80. doi: 10.3390/genes9020080 (PMC5852576; doi:10.3390/genes9020080)
Supplement: Supplementary file 1 [file genes-09-00080-s001.zip › Table S2.docx]

| Gene | GenBank Accession number |
| --- | --- |
| *dfrD* | U43152 |
| *dfrG* | JX120102.1 |
| *ermA* | KC456362 |
| *ermB* | JX535233 |
| *ermC* | GQ483470.1 |
| *fepR* | KJ000253 |
| *fosX* | WP_003726635 |
| *gyrB* | NC_03211 |
| *gyrA* | NC_03210 |
| *oatA* | NC_003210 |
| *parE* | NC_03210 |
| *parC* | NC_03210 |
| *spc* | NC_021513 |
| *tetA* | NC_022568 |
| *tetL* | NC_05013.1 |
| *tetM* | U09422 |
| *tetS* | NG_048273 |
| *vanA* | NC_013317 |
| *vanB* | NC_021994 |
